# Supplementary material for: Virulence Reduction in Yersinia pestis by Combining Delayed Attenuation with Plasmid Curing
Source: Biomolecules. 2025 Dec 25;16(1):40. doi: 10.3390/biom16010040 (PMC12839094; doi:10.3390/biom16010040)
Supplement: Supplementary file 1 [file biomolecules-16-00040-s001.zip › biomolecules-4027845-supplementary.pdf]

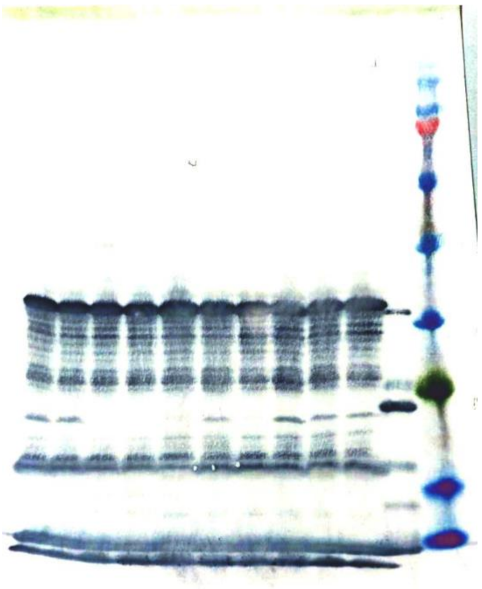

Line 1 - wild type *Y. pestis* strain 231 grown in BHI without 0.2% arabinose
Line 2 - wild type *Y. pestis* strain 231 grown in BHI with 0.2% arabinose
Line 3 - *Y. pestis* strain 231Δ*crp* grown in BHI without 0.2% arabinose
Line 4 - *Y. pestis* strain 231Δ*crp* grown in BHI with 0.2% arabinose
Line 5 - *Y. pestis* strain 231 P_{BAD}-*crp* # 1 grown in BHI without 0.2% arabinose
Line 6 - *Y. pestis* strain 231P_{BAD}-*crp* # 1 grown in BHI with 0.2% arabinose
Line 7- *Y. pestis* strain 231 P_{BAD}-*crp* # 2 grown in BHI without 0.2% arabinose
Line 8- *Y. pestis* strain 231P_{BAD}-*crp* # 2 grown in BHI with 0.2% arabinose
Line 9 - wild type *Y. pestis* strain 231 grown in BHI without 0.2% arabinose
Line 10 - wild type *Y. pestis* strain 231 grown in BHI with 0.2% arabinose
Line 11 - recombinant Crp
Line 12 – ColorMixed Protein Marker 180 (ABclonal, EC) (180 kDa, 140 kDa,100 kDa, 75 kDa,60 kDa,45 kDa,35 kDa,25 kDa,15 kDa,10 kDa)
